# Capitalizing on Shared Interests: Achieving Age-Friendly University Endorsement Through Partnership and Advocacy

**DOI:** 10.1093/geroni/igaa057.1751

**Published:** 2020-12-16

**Authors:** Katherina Terhune, Allyson Graf, Suk-Hee Kim, Amy Danzo, Sara Conwell

**Affiliations:** Northern Kentucky University, Highland Heights, Kentucky, United States

## Abstract

Response to the lifelong educational needs and interests of the older adult population calls for new opportunities and innovative practices in teaching, research, and community engagement that universities need to be prepared to offer their students and community. This presentation provides information from an Age-Friendly University (AFU) that currently has no gerontology degree or certificate program, highlighting various campus wide programs and initiatives aligned with the AFU principles. In particular, collaborative strategies involving faculty and the Adult Learner Programs and Services to help promote campus buy-in and AFU endorsement will be emphasized. Practical strategies will be discussed to assist junior and senior level faculty and other university personnel wanting to achieve AFU endorsement at their institution of higher education in order to shape age-friendly programs and practices.

